# Association Between Myocardial Scar Burden and Left Ventricular Ejection Fraction in Ischemic Cardiomyopathy

**DOI:** 10.7759/cureus.12110

**Published:** 2020-12-16

**Authors:** Fatma Aboul Enein, Sarah Allaaboun, Samiha Khayyat, Mariam Andijani, Mazen M Alkhuzai, Aseel A Aljunied, Magdi Al Adhreai

**Affiliations:** 1 Cardiology, Alexandria University Faculty of Medicine, Alexandria, EGY; 2 Cardiology, King Abdullah Medical City, Mecca, SAU; 3 Cardiology, Faculty of Medicine, Umm Al-Qura University, Mecca, SAU

**Keywords:** ischemic cardiomyopathy, viability, cad, prognosis, mri cardiac, heart failure with reduced ejection fraction

## Abstract

Background

This study was conducted to assess the relationship between scar burden (extent and severity) and the follow-up left ventricular ejection fraction (LVEF).

Methods

Patients were referred for viability assessment with late gadolinium enhancement (LGE) on cardiovascular magnetic resonance imaging. To measure the transmural extent of LGE in each segment (scar score), we used a five-point scale system. Baseline ejection fraction (EF) and at follow-up were recorded. LVEF classified as non-severe and severely depressed.

Results

The study included 178 patients (males: 88.8%; mean age: 57.1±10.02 years; mean baseline LVEF: 28.61±10.39). In patients with severe baseline LVEF, the mean scar percentage was higher than that in patients who had non-severe LVEF (38.8±19.41 vs. 24.61±21.21; p˂0.001). On linear regression analysis, aldosterone antagonist and total scar score significantly predicted follow-up ejection fraction (EF) (B=-7.083, p˂0.001 and B=-3.038, p=0.038, respectively). Left anterior descending artery (LAD) territory viability and baseline EF significantly predicted change in EF in patients with LVEF ≤ 35% (B=5.389, p=0.009 and B=-0.581, p˂0.001, respectively). On binary regression analysis for the prediction of at least 5% improvement in EF in patients with baseline EF ≤ 35%, baseline EF and LAD viability were significant (B=-0.15, p=0.014 and B=1.042 and p=0.054, respectively).

Conclusions

The extent of myocardial scar and viability of LAD territory are identified as the important and independent parameters for the predictions of improvement in EF even after adjustment for demographics and baseline EF and following the standards of care medication.

## Introduction

Magnitude of the problem

Currently, more than five million patients have been diagnosed with heart failure (HF), with the figures still rising and over 650,000 new cases diagnosed annually [1]. Although the survival of HF patients have improved, the absolute mortality rates of HF patients remains 50% if diagnosed within five years of its occurrence [2]. Coronary artery disease (CAD) with a history of myocardial infraction (MI) is a major cause of HF with reduced ejection fraction (EF) and ischemic cardiomyopathy (ICM). [3] It is estimated that CAD may cause more than 11 million deaths globally [4] in the next 20 years [5]. Mortality rates in ICM patients with severely depressed EF are significantly higher than the general patient [6]. Despite significant advancements in medical and device therapies, outcomes in severe HF are non-satisfactory.

Cardiac magnetic resonance imaging (CMR)

For the evaluation of viability and subsequent effects of clinical management plan, cardiac magnetic resonance imaging (CMR) is the gold standard, and using a paramagnetic contrast agent (gadolinium), scar can be identified using as late gadolinium enhancement (LGE). With increase in extracellular space due to fibrosis, the gadolinium-based contrasts wash out slowly from the myocardium, which enhances areas of scarring. Scarring may be observed in both ischemic and non-ischemic cardiomyopathy; the former extends from the subendocardium to the epicardium and is matched to the area of infraction related artery [7]. Myocardial infarction (MI) causes scar in the myocardium, which affects the left ventricle (LV) contractile function and results in the reduction of systolic function.

CMR is useful in exploring the correlation between size of myocardial scar, localization, and transmurality of long-term LV remodeling of healed MI patients [8]. The quantity and transmural extent of myocardial scar tissues on LGE are predictors of mortality in patients with CAD that were independent from reduced LVEF [9].

The aim of this study is twofold: firstly, to investigate the relation between scar burden (extent and severity) and follow-up EF in ICM, and, secondly, to identify predictors of EF improvement.

## Materials and methods

After local Institutional Review Board approval, we identified 500 consecutive patients who had undergone CMR for viability assessment from 2012 to 2018. Patients with non-CAD cardiomyopathies were not included in this study. Data were collected from the electronic medical files. Data recorded included demographics, medication, echocardiography, coronary angiogram, and CMR results. Medications included β-blockers, angiotensin-converting enzyme inhibitors (ACE-I), spironolactone, and statins. Post-CMR study coronary revascularization (either percutaneous or surgical) was also recorded.

Echocardiography protocol

The American Society of Echocardiography guidelines were followed to review echocardiogram reports. LVEF was classified as follows: non-severe LVEF was defined as LVEF ˃ 35%, and severely abnormal was defined as LVEF ≤ 35%. In patients with follow-up echocardiography, LVEF was calculated using the Simpson biplane method by an echocardiographer (F. A.) blinded from clinical analysis. An improvement in LVEF ≥ 5% was used to define global functional recovery from responders [10].

CMR protocol and analysis

CMR study was conducted using a MAGNETOM Espree 1.5-Tesla MRI (Siemens, Malvern, PA, USA). CMR images were evaluated by an experienced cardiologist with level II certified training (F. A.) blinded from clinical analysis. Semiquantitative analysis was performed using a 17-segment model [11]. To define the transmural extent of LGE in each segment, a five-point scale was followed, where the scar score was given as follows: 0 = no LGE; 1 = 1%-25% LGE; 2 = 26%-50% LGE; 3 = 51%-75% LGE; and 4 = 76%-100% LGE. Transmural extent of a segment with LGE 1-50% was considered as “viable” and with LGE 51-100% as “scar” [12]. Based on scar transmurality, each segment was scored. LV scar score (LVSS) was calculated as a sum of the scores of all the segments dividing by 17. LVSS of 0 represents no scar. To know the extent of scar tissues, quantitative analysis was performed on the basis of following parameters: viable segments with a scar score of 0, 1, or 2, and nonviable segments with a scar score of 3 or 4.

Statistical analysis

SPSS Version 21 (IBM Corp., Armonk, NY, USA) was used for the statistical analysis of data. Continuous data were expressed as mean ± SD and compared using Student's t-test. Categorical data were expressed as percentages and compared using a chi-square test. Regression models were used, and variables with a p-value of <0.05 as tested on univariate analysis were incorporated into the multivariate models as continuous or dichotomous variables. A p-value of <0.05 was considered statistically significant.

## Results

Table 1 summarizes the baseline characteristics, coronary angiographic results, CMR results, and medication. A total of 178 consecutive patients were referred for CMR viability, with an average age of 57.1±10.0 years and with a mean baseline LVEF% of 28.6±10.4. Of the patients, 88.8% were males; 67.5% had multivessel disease, and in 64.6% the left anterior descending artery (LAD) territory was non-viable. Patients were categorized into group based on their baseline LVEF: group I (LVEF ≤ 35%) and group II (LVEF > 35%). Most of patients in group I had multivessel disease (63.1%) as compared to group II (44.1%) (p=0.02). Regarding change in EF (follow-up EF and baseline EF) there was no significant difference between the two groups. A total of 42 patients underwent revascularization, of those 38% demonstrated improvement in LVEF.

**Table 1:** Baseline characteristics based on severity of EF EF, ejection fraction; BMI, body mass index; HTN, hypertension; DM, diabetes mellitus; ACE-I, angiotensin-converting enzyme inhibitors; LAD, left anterior descending artery; LVEF, left ventricular ejection fraction

	Whole Cohort (n=178)	Group I: EF ≤ 35% (n=144)	Group II: EF > 35% (n=34)	p-Value
Clinical data				
Male, n (%)	158 (88.8)	132 (91.7)	26 (76.4)	0.012
Age, years, mean±SD	57.1±10.02	57.0±9.95	57.4±10.5	0.83
BMI, mean±SD	28±5.2	27.8±5.2	28.9±4.9	0.225
HTN, n (%)	118 (66.3)	99 (68.8)	19 (55.8)	0.153
DM, n (%)	123 (69.1)	104 (72.2)	19 (55.8)	0.064
Smoking, n (%)	35 (19.7)	23 (15.9)	12 (35.2)	0.012
Baseline EF (%)	28.6±10.4			
Number of significantly diseased coronaries				
Single-vessel disease, n (%)	27 (17.2)	17 (11.8)	10 (29.4)	0.02
Two-vessel disease, n (%)	19 (12.1)	17 (11.8)	2 (5.8)	
Three-vessel disease, n (%)	106 (67.5)	91 (63.1)	15 (44.1)	
Medications and revascularization				
Aspirin, n (%)	174 (97.8)	140 (97.2)	34 (100)	0.326
Statin, n (%)	162 (91)	131 (90.9)	31 (91.1)	0.97
β-blocker, n (%)	176 (98.9)	143 (99.3)	33 (97)	0.264
ACE-I, n (%)	136 (76.4)	115 (79.8)	21 (61.7)	0.025
Aldosterone antagonist, n (%)	102 (57.3)	94 (65.2)	8 (23.5)	<0.001
Revascularization, n (%)	42 (23.6%)	35 (24.3%)	7 (20.5%)	0.646
Imaging results				
Baseline EF (%)	28.6±10.4			
Number of non-viable segment, mean±SD	3.76±3.2	4.09±3.18	2.35±3.01	0.004
Segment %, mean±SD	36.1±20.5	38.8±19.41	24.61±21.21	<0.001
LAD territory				
Viable, n (%)	63 (35.4)	41 (28.4)	22 (94.1)	<0.001
Non-viable, n (%)	115 (64.6)	103 (71.5)	12 (35.2)	
Follow-up echocardiography				
Change in EF%	4.9±9.4	5.5±9.7	2.35±6.7	0.207
LVEF improvement of ≥5%, n (%)	58 (32.6)	49 (34)	9 (26.4 )	0.39
LVEF improvement of >5% in revascularization subgroup	16 of 42 (38%)	15 of 35 (42%)	1 of 7 (14%)	0.155

Table 2 summarizes predictors of changes in EF in all patients using linear regression analysis. Significant predictors identified in univariable analysis were hypertension, diabetes mellitus (DM), aldosterone antagonist, total scar score, and LAD territory. However, in multivariable analysis, only aldosterone antagonist and the LVSS were found to be significant after adjusting for LAD territory viability. An interaction was observed between the LAD viability and total scar score.

**Table 2:** Linear regression analysis for the predictors of change in EF in whole cohort HTN, hypertension; DM, diabetes mellitus; ACE-I, angiotensin-converting enzyme inhibitors; LVSS, left ventricular scar score; LAD, left anterior descending artery; EF, ejection fraction

	Univariable Analysis			Multivariable Analysis		
Factor	Odd’s ratio	p-Value	95% CI	Odd’s ratio	p-Value	95% CI
Age	-0.003	0.976	-0.23 to 0.23			
HTN	-5.654	0.014	-10.16 to -1.15	-3.911	0.144	-9.18 to 1.36
DM	-4.944	0.038	-9.68 to -0.29	-1.047	0.704	-6.49 to 4.40
Smoking	-3.133	0.184	-7.83 to 1.56			
Statin	0.150	0.968	-6.32 to 7.62			
ACE-I	-2.980	0.277	-8.38 to 2.43			
Aspirin	4.147	0.512	-8.37 to 16.66			
Aldosterone antagonist	-8.088	0.000	-12.13 to -4.04	-7.083	0.000	-10.95 to -3.22
Revascularization	2.59	0.28	-2.12 to 7.81			
Number of vessels	-2.24	0.07	-4.63 to 0.16			
LVSS	-4.15	0.003	-6.85 to -1.46	-3.038	0.038	-5.9 to -0.2
Scar percentage	-0.17	0.003	-0.27 to -0.09			
Transmurality	-0.85	0.011	-1.50 to -0.200			
LAD territory viability	6.39	0.003	2.19 to 10.61	3.488	0.126	-1.0 to 7.98
Number of viable segments	0.852	0.011	0.200 to 1.503			

Table 3 shows linear regression analysis for the predictors of change in EF in group I (baseline EF ≤ 35%). Only baseline EF and presence of LAD viability were independent predictors for change in EF on follow-up.

**Table 3:** Linear regression analysis for the predictors of change in EF in group I (baseline EF ≤ 35%) DM, diabetes mellitus; ACE-I, angiotensin-converting enzyme inhibitors; LAD, left anterior descending artery; EF, ejection fraction

Factor	B	p-Valve	95% CI
Age	0.049	0.648	-0.163 to 0.260
DM	-3.340	0.150	-7.915 to 1.235
ACE-I	-0.300	0.911	-5.651 to 5.050
Aldosterone antagonist	-2.200	0.326	-6.360 to 2.231
LAD territory viability	5.389	0.009	1.407 to 9.371
Baseline EF	-0.581	0.000	-0.861 to -0.302

Table 4 shows multivariable binary regression analysis for the prediction of at least 5% improvement in EF in patients with baseline EF ≤ 35% (group I); only baseline EF and presence of LAD viability were independent predictors for improvement of EF on follow-up.

**Table 4:** Multivariable binary regression analysis for the prediction of at least 5% improvement in EF in patients with baseline EF ≤ 35% (group I) DM, diabetes mellitus; LAD, left anterior descending artery; EF, ejection fraction

Factor	B	p-Valve	95% CI
Age	0.023	0.402	0.970 - 1.078
DM	-1.121	0.072	0.096 - 1.106
Aldosterone antagonist	-0.980	0.108	0.113 - 1.241
LAD territory viability	1.042	0.054	0.981 - 8.190
Baseline EF	-0.150	0.014	0.828-0.979

## Discussion

The objective of this study was to identify the prognostic role of role of scar burden in ICM. Our results revealed that the follow-up LVEF is influenced by scar burden, as evident by total scar scores. Furthermore, we observed that LAD viability is an independent predictor of follow-up LVEF, and conversely revascularization did not affect follow-up LVEF.

Pathophysiology of myocardial infraction and hibernation

Myocardium may exist in different physiological states, namely normal, hibernating, and nonviable. First observed changes (10-15 minutes after the onset of ischemia) are loss of cellular glycogen, myofibrils relaxation, and disruption in sarcolemma. Using electron microscopy, mitochondrial abnormalities were noted after 10 minutes of coronary occlusion and they were progressive [13]. Progression in necrosis from the subendocardium to subepicardium occurs over several hours. Collateral flow, myocardial oxygen consumption, and intermittent occlusion/reperfusion are the important factors affecting precondition of the heart [14].

Histopathological changes of scar by CMR

Hibernating myocardium is dysfunctional, viable, and capable of recovering the function after reperfusion [15]. Gunning et al. in a study on CMR and myocardial biopsy showed that the hibernating myocardium contain myocyte content that is similar to normally perfused myocardium but it was significantly higher than the scar areas [16]. Ventricular biopsies taken at coronary artery bypass grafting (CABG) reported morphological changes characterized by myocyte de-differentiation and loss of sarcomeres, sarcoplasmic reticulum, and T-tubules (contractile apparatus) [17].

Despite the preserved numbers, histopathological variations were associated with abundance in glycogen deposition, rough endoplasmic reticulum strands, and reduced consumption of mitochondrial oxygen. These changes were perfusion dependent, more in endocardium, and directly correlate with the severe condition of stenosis in subtending coronary artery [17]. Research studies support the model of hibernation as the chronic adaptive response, which can eventually become irreversible [18]. Regression in glycogen accumulation and restoration of myofibril content could be possible; however, the myocardial fibrosis is not reversible [16].

Hibernating myocardium and contractile improvement

In a study of the timeline of contractile function improvement in hibernating myocardium, 31% of the hibernating segments improved at third month and 61% showed additional recovery at 14 months [19]. This is in agreement with our findings, where 58 patients (32.5%) showed a >5% improvement in LVEF; however, only 42 (23.6%) had revascularization (percutaneous coronary intervention [PCI] or CABG). The lower frequency of revascularization may be in part attributed to patient or surgeon decision. The result could be explained in part by the ORBITA trial, where patients on optimal medical treatment had similar outcomes as those on PCI [20].

CMR scar extent and functional improvement

Myocardial viability is pivotal to the clinical decision plan in managing ICM. Assessment of scar extent and/or fibrosis and its size using LGE-CMR is accurate and reproducible. Several studies have been conducted to estimate the extent, and LGE amount as an important parameter for an independent prediction of functional recovery and outcome. With LGE, one can predict the major adverse cardiac events and the mortality rate beyond the coronary anatomy or clinical factors, which is independent of EF [21]. The transmurality of scar is inversely related to functional recovery. Segments with >50% scar (non-viable) have a low probability of functional recovery [22]. The presence of ≥10 segments with viability predicts a global functional recovery [23]. The presence of ≤4 scar segments (25% of LV) is a close-off limit to predict LVEF improvement [24]. This is in an agreement with the findings of our study where the total scar score was observed as an independent predictor of LVEF improvement after an adjustment for DM and other medications. Remodeling of LV with large ended systolic volume can prevent the global recovery, even in the patients with substantial viability. Improvement of LVEF may be observed within one year of post-revascularization [19].

Ischemic cardiomyopathy: optimal medical treatment and prognosis

Medical treatment of the patients with HF has improved substantially with the introduction of ACE-I, angiotensin II receptor blockers, spironolactone, and β-blockers. However, in patients with severe HF, the mortality rate remains high. Survival rate estimates were 50% and 10% at 5 and 10 years, respectively [25]. After CABG, contractile function improves in one-third segments and LVEF improves in only 40% patients [26]. In clinical practices, LVEF is important for patient prognostics. Hence, improved LVEF may affect prognosis, at least in parts. Identification of patients with potential to improve LVEF is important for survival, risk stratification, and clinical management plan. A recent meta-analysis of 5,286 patients concluded that the optimal medical strategy is comparable to revascularization strategy regarding prognosis, death, MI, repeat revascularization, or angina [27]. This was the management strategy taken by the majority of patients observed during our study, and only one-third underwent revascularization.

Impact of scar and disease progression

LVEF is considered a strong predictor of sudden cardiac death among the clinical observations, but delay in hyperenhancement MRI may recognize myocardial scar, which is a substrate of developing a probably fatal ventricular arrhythmia. Klem et al. [28] also observed a strong prognostic ability of myocardial scar burden for the prediction of adverse outcomes, which suggests that quantification of scar was superior to LVEF ≤ 30% to recognize high-risk patients. They studied 137 patients, out of which 53% were with ICM and 25/65 patients with LVEF ≤ 30% died or had an appropriate implantable cardioverter-defibrillator (ICD) discharge at the time of follow-up. Patients with scar ≤ 5% were having event rate below or similar to entire group with LVEF > 30% and were at a low risk. Furthermore, Gao et al. [29] reported that the scar observed through the CMR provides a prediction on arrhythmic events in patients assessed for ICD implantation. However, it is important to note that both the studies considered non-ICM as well as ICM patients.

Role of revascularization

In this study, 42 (23.6%) patients had undergone revascularization by either PCI or CABG after CMR. A non-significant difference was noted in the rate of LVEF improvement and the scar score. The role of revascularization in ICM is still not determined. Gerber et al. have reported that the survival rate was significantly worse when there was no revascularization of dysfunctional viable myocardium [30]. A multicenter study called the HF Revascularization Trial (HEART) was performed on 800 ICM patients and explained that the conservative management strategy may not be inferior to revascularization [18].

Clinical relevance

LVEF is well accepted as one of the strong predictors of sudden cardiac death; CMR is the gold standard for identifying myocardial scar and viability. An important aspect of our study is quantifying scar burden as determined by the magnitude of LGE, hence giving a global view of the heart rather than focusing on viability in a binary fashion. Our data reveal that the quantification of scar burden could offer more risk stratification in ICM patients and may identify the cases with more disease progression even after optimal medical treatment and those who may be candidates for device implantation.

## Conclusions

In ICM, myocardial scar burden and viability of LAD territory are independent prognostic predictors even after adjustment for demographics, baseline EF, and medications.
